# TNF receptor family signaling in the development and functions of medullary thymic epithelial cells

**DOI:** 10.3389/fimmu.2012.00278

**Published:** 2012-09-04

**Authors:** Taishin Akiyama, Miho Shinzawa, Nobuko Akiyama

**Affiliations:** Division of Cellular and Molecular Biology, Department of Cancer Biology, Institute of Medical Science, University of TokyoTokyo, Japan

**Keywords:** medullary thymic epithelial cells, TNF receptor family, NF-κB, signal transduction, self-tolerance, autoimmune disease

## Abstract

Thymic epithelial cells (TECs) provide the microenvironment required for the development of T cells in the thymus. A unique property of medullary thymic epithelial cells (mTECs) is their expression of a wide range of tissue-restricted self-antigens, critically regulated by the nuclear protein AIRE, which contributes to the selection of the self-tolerant T cell repertoire, thereby suppressing the onset of autoimmune diseases. The TNF receptor family (TNFRF) protein receptor activator of NF-κB (RANK), CD40 and lymphotoxin β receptor (LtβR) regulate the development and functions of mTECs. The engagement of these receptors with their specific ligands results in the activation of the NF-κB family of transcription factors. Two NF-κB activation pathways, the classical and non-classical pathways, promote the development of mature mTECs induced by these receptors. Consistently, TNF receptor-associated factor (TRAF6), the signal transducer of the classical pathway, and NF-κB inducing kinase (NIK), the signal transducer of the non-classical pathway, are essential for the development of mature mTECs. This review summarizes the current understanding of how the signaling by the TNF receptor family controls the development and functions of mTEC.

## Introduction

The development of self-tolerant T cells and regulatory T cells in the thymus requires a microenvironment of both non-hematopoietic stroma cells and cells of hematopoietic origin (Gill et al., 2003; Takahama, 2006; Anderson and Takahama, 2012). Thymic epithelial cells (TECs), essential components of this unique microenvironment, are subdivided into two major subtypes according to their localization: medullary thymic epithelial cells (mTECs) and cortical thymic epithelial cells (cTECs). IL-7 and Delta-like 4 expressed by TECs promote their proliferation and commitment to the T-cell lineage, thereby leading to the differentiation of CD4^+^CD8^+^ double-positive (DP) T cells expressing a diverse repertoire of T-cell antigen receptors (TCRs) (Hozumi et al., 2008; Koch et al., 2008; Hong et al., 2012). Subsequently, this T-cell repertoire is scrutinized by the TECs displaying the complex of self-peptides and MHC molecules (self-pMHCs) (Kyewski and Klein, 2006; Anderson et al., 2007; Klein et al., 2009). The presentation of self-pMHCs by cTECs is required for the survival and differentiation of DP cells to CD4- or CD8-single-positive (CD4SP or CD8SP) T cells. This process, positive selection, is achieved when a TCR binds to a self-pMHC with moderate avidity, whereas T cells expressing a TCR recognizing a self-pMHC with high avidity undergo apoptosis, i.e., negative selection. Several studies suggest that mTECs are involved in negative selection, thereby preventing the onset of autoimmune diseases (Mathis and Benoist, 2004; Kyewski and Klein, 2006; Anderson et al., 2007; Klein et al., 2009). Moreover, mTECs might contribute to the development of thymic regulatory T cells (Tregs) expressing the transcription factor Foxp3 (Hsieh et al., 2012), which suppress autoimmune responses and excessive immune reactions (Wing and Sakaguchi, 2010; Josefowicz et al., 2012). Given the connections with autoimmune diseases, researchers have been striving to uncover the molecular mechanisms underlying the functions and development of mTECs. In this review, we primarily focus on the role of signaling by the TNF receptor family (TNFRF) members RANK, CD40, and LtβR in the development and functions of mTECs in the establishment of self-tolerance.

## Functions of mTECs

**Figure 1:**
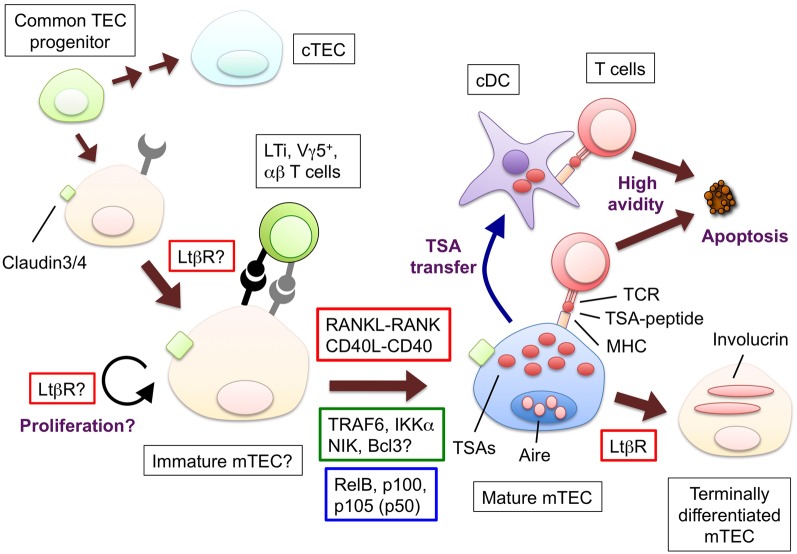
**The hypothetical model for the development and functions of mTECs and molecules to promote the mTEC development.** Bipotent common progenitors give rise to the mTEC progenitors expressing claudin-3 and -4. Lymphoid tissue inducer (LTi), Vγ5^+^ DETC progenitor (Vγ5^+^) or positively selected α β T cells produce RANKL; CD40L is supplied only by α β T cells. The interaction between RANKL and RANK promotes the differentiation and/or proliferation of the mTECs. The similar contribution of the interaction of CD40L and CD40 begins at the neonatal stage. RANK and CD40 signaling results in the translocation of the NF-κB family member RelB through the activation of signal transducers TRAF6 and NIK. The mature mTECs express a wide variety of tissue-specific antigens (TSAs) and AIRE. The TSAs are in part transferred to conventional DCs; as a result, both the mTECs and DCs would display the TSA-peptides to the developing T cells. The T cells expressing TCR bound to the complex of the TSA-peptide and MHC molecules with high avidity undergo apoptosis. Mature mTECs might further differentiate into involucrin-expressing mTECs in the LtβR and AIRE-dependent process. The LtβR signaling might regulate the up-regulation of RANK in the TECs and/or enhance the proliferation of the RANK-expressing TECs. Ligands and receptors involved in each process are surrounded by red line rectangles, signal transducers are by green line rectangles, transcription factors are by blue line rectangles.

It is well established that mTECs ectopically express a wide variety of self-antigens that are normally expressed in a tissue-specific fashion (TSAs, for example, insulin, c-reactive protein, and caseins) (Kyewski and Klein, 2006). Because mature mTECs express high levels of MHC class II, co-stimulatory molecule CD80 and antigen-processing enzymes (Gray et al., 2006; Guerder et al., 2012), it is likely that mTECs directly perform negative selection by presenting TSA-peptides, as was confirmed in a recent study (Hinterberger et al., 2010) (Figure 1). Additionally, the TSAs expressed in mTECs are transferred to and presented by cDCs for negative selection (Gallegos and Bevan, 2004; Koble and Kyewski, 2009) (Figure 1). Consequently, the T cells that are potentially reactive to the TSAs in the periphery would undergo apoptosis in the thymus. It is important future study to clarify the relative contributions of mTECs and cDCs to negative selection.

Autoimmune regulator (AIRE), for which loss of function mutations cause an inherited human autoimmune disease (Consortium, 1997; Nagamine et al., 1997; Michels and Gottlieb, 2010), is expressed in mTECs and promotes TSA expression, thereby suppressing the onset of autoimmune diseases (Anderson et al., 2002; Peterson et al., 2008; Mathis and Benoist, 2009). Although the involvement of AIRE is evident, the molecular mechanisms underlying a broad range of TSA expression in mTECs remain unclear. The unique epigenetic regulation in mature mTECs appears to regulate the diversity of TSA expression (Derbinski et al., 2005; Tykocinski et al., 2010). Interestingly, individual mTECs appear to have a different and transient TSA expression pattern, implying the probabilistic nature of the expression of TSAs (Derbinski et al., 2008; Villaseñor et al., 2008).

The requirement of TSA expression in preventing the onset of autoimmune diseases was suggested by the results of several studies (Kyewski and Klein, 2006). It was reported that the polymorphisms in the promoter region of the muscle acetylcholine receptor gene (*CHRNA1*), which is associated with the early onset of human autoimmune myasthenia gravis, leads to the reduction of *CHRNA1* expression in human mTECs (Giraud et al., 2007). Two studies provide more direct evidence for the correlation between the expression of TSAs in mTECs and autoimmunity in the corresponding tissues. The lack of interphotoreceptor retinoid-binding protein in thymic stroma cells is sufficient for inducing spontaneous autoimmunity in the eyes (DeVoss et al., 2006). Moreover, deletions of insulins in AIRE-expressing mTECs spontaneously provoked autoimmune diabetes in mice (Fan et al., 2009).

The high avidity interaction between TCRs and self-pMHCs is the determinant of the development of Treg (Hsieh et al., 2012; Josefowicz et al., 2012). Previous studies revealed that the cTEC-specific expression of MHC class II (MHC II) is sufficient for Treg generation in the thymus (Bensinger et al., 2001). Moreover, a recent study revealed that the diversity of TCRs in Tregs was not altered by the lack of AIRE (Daniely et al., 2010). These data suggest that TSA presentation by mTECs is dispensable for thymic Treg development. However, almost all Foxp3^+^ cells in the thymus are CD4SP T cells localized in the medulla (Lee and Hsieh, 2009), and Foxp3^+^ expression initiates at the stage of the CD4SP progenitor (Burchill et al., 2008; Lio and Hsieh, 2008). Thus, the contribution of mTECs to Treg differentiation and/or proliferation cannot be ruled out (Hsieh et al., 2012). Indeed, several mTEC-deficient mice showed a partial reduction in the Treg frequency in the thymus (Kajiura et al., 2004; Shimo et al., 2011).

The chemokines CCL19 and CCL21 secreted from mTECs attract positively selected T cells expressing the chemokine receptor CCR7 into the medulla (Ueno et al., 2002, 2004). A recent study revealed that mTECs also express the chemokine XCL to attract cDCs in the medulla (Lei et al., 2011). Interestingly, the expression of CCL19, CCL21, and XCL appear to be regulated by AIRE (Laan et al., 2009; Lei et al., 2011). These findings might be consistent with the idea that AIRE regulates the differentiation of mTECs as well as the TSA expression (Gillard et al., 2007; Dooley et al., 2008; Yano et al., 2008).

## Development of mTECs

The endodermal epithelial cells in the ventral part of third pharyngeal pouch differentiate into TECs (Blackburn and Manley, 2004; Holländer et al., 2006). Then, both the mTECs and cTECs are differentiated from bipotent TEC progenitors (Bleul et al., 2006; Rossi et al., 2006). The existence of mTEC-committed progenitors was initially proposed from the finding that the mTECs form clusters expressing a single MHC II in the thymuses of chimeric mice with two different types of MHC II molecules (Rodewald et al., 2001). Furthermore, it was shown that the TEC fraction expressing claudin-3 and -4, components of tight junction, differentiates into AIRE^+^ mTECs but not into cTECs (Hamazaki et al., 2007). Another study suggested that CD80^−^ UEA-1-lectin^+^ TECs emerging in embryonic thymus contain the mTEC progenitors (Gäbler et al., 2007). The rapid turnover of mTECs (Gray et al., 2006, 2007) and post-mitotic nature of the AIRE-expressing cells (Gray et al., 2007) in the adult thymus imply the presence of progenitors that continuously provide mature mTECs. Indeed, the CD80^−^ UEA-1^+^ TECs prepared from postnatal thymuses are converted into CD80^+^ UEA-1^+^ mTECs in re-aggregated fetal thymus organ cultures (Gäbler et al., 2007). Currently, the ratio of mTEC progenitors in the claudin-3/4^+^ CD80^−^ UEA-1^+^ TEC fraction is unclear, and it remains elusive whether the mTEC progenitors in the adult thymus are identical to those in the embryonic thymus.

In the adult thymus, the mTECs are separated into mainly two subsets (Gray et al., 2006). The first subset expresses relatively higher levels of MHC II and CD80, usually referred to as mTEC^high^; this subset contains AIRE-expressing cells and is regarded as the mature type (Kyewski and Klein, 2006). The other subset, mTEC^low^, shows relatively lower expression levels of MHC II and CD80. The mTEC^high^ subset would be expected to play a role in negative selection because of the expression of AIRE, the wide range of TSAs as well as their high antigen-presenting ability, whereas the contribution of the mTEC^low^ subset on thymic tolerance remains to be addressed. In addition to these two subsets, the mTECs expressing involucrin, a keratinocyte terminal differentiation marker, are regarded as terminally differentiated mTECs (Yano et al., 2008; White et al., 2010). Moreover, a recent fate mapping of AIRE expression suggested another developmental stage of mTECs in which the expression of AIRE ceases (Nishikawa et al., 2010).

## TNF receptor family signaling

The binding of TNF family ligands to the TNFRF proteins leads to various cellular responses, such as proliferation, differentiation, inflammatory responses, and apoptosis (Aggarwal, 2003; Hehlgans and Pfeffer, 2005; Aggarwal et al., 2012). Several members of the TNFRF activate NF-κB family transcription factors, inducing the expression of the genes required for these cellular responses (Aggarwal, 2003; Hehlgans and Pfeffer, 2005; Vallabhapurapu and Karin, 2009). Several lines of evidence have indicated that the signaling of the TNFRF members RANK, CD40, and LtβR play critical roles in the development and function of mTECs.

## CD40L-CD40

The engagement of CD40 with CD40L initiates and progresses acquired immune responses through the activation and survival of B cells, macrophages, and DCs (Elgueta et al., 2009; Ma and Clark, 2009). The expression of CD40 in human TECs was initially reported almost two decades ago (Galy and Spits, 1992), and, because the CD40 signal exerts profound effects on antigen presenting cells, several studies on the function of CD40 in the thymus have focused on the roles in proliferation and the selection of T cells (Foy et al., 1995; Ruggiero et al., 1996; Williams et al., 2002) and Treg development (Kumanogoh et al., 2001; Spence and Green, 2008; Martín-Gayo et al., 2010).

Several studies have provided evidence that CD40 signaling controls the development of mTECs. The forced expression of CD40L by the lck promoter caused a reduction of the cTECs and an increase of mTECs (Dunn et al., 1997), and both CD40L-deficient (CD40L-KO) mice and CD40-KO mice showed a reduction of mTECs (Gray et al., 2006; Akiyama et al., 2008; White et al., 2008). In addition, the *in vitro* development of mTECs is elicited by the addition of recombinant CD40L protein to fetal thymic stroma organ culture (Akiyama et al., 2008), which is prepared from fetal thymus by the elimination of cells of hematopoietic origin. Notably, whereas CD40 is expressed in the TECs of fetal thymus (Dunn et al., 1997; Akiyama et al., 2008; Shakib et al., 2009), the expression of CD40L was not detected in the fetal thymus but only began in the neonatal mouse (Dunn et al., 1997; Akiyama et al., 2005). This expression pattern suggested that the contribution of the CD40L-CD40 interaction to the development of mTECs should be limited following birth. The expression of CD40L in the thymus was predominantly detected in the medulla (Dunn et al., 1997). Consistently, some studies indicated that the expression of CD40L is up-regulated in positively selected CD4SP T cells (Hikosaka et al., 2008; Irla et al., 2008).

## RANKL-RANK

The RANKL and RANK interaction regulates a diverse set of physiological events, such as bone homeostasis (Yasuda et al., 1998; Dougall et al., 1999; Kong et al., 1999), lymph node development (Kong et al., 1999; Kim et al., 2000), mammary gland development (Fata et al., 2000), hair follicle anagen (Duheron et al., 2011), and DC survival (Anderson et al., 1997; Wong et al., 1997). RANKL-deficient (RANKL-KO) and RANK-deficient (RANK-KO) mice exhibited a reduction in mature mTECs (Rossi et al., 2007; Akiyama et al., 2008; Hikosaka et al., 2008), which was more remarkable for the mTEC^high^ subset. Moreover, recombinant RANKL protein efficiently induces the development of mTECs expressing AIRE and TSAs in fetal thymic stroma culture (Rossi et al., 2007; Akiyama et al., 2008). Although a lack of the RANKL-RANK interaction causes a significant reduction of mature mTECs, a small population of mature mTECs still exists in these mutant mice (Akiyama et al., 2008; Hikosaka et al., 2008), a finding that suggests the presence of another signal for inducing mature mTECs in addition to RANK signaling. Indeed, mature mTECs were completely absent in RANKL- and CD40-double deficient (RANKL/CD40 DKO) mice (Akiyama et al., 2008). Thus, RANK and CD40 signaling have an overlapping function in the development of mature mTECs. Osteoprotegerin, OPG, a natural inhibitor of RANKL, is expressed in mTECs (Hikosaka et al., 2008), and, consistently, OPG-deficient mice showed an increase in mTECs in the thymus (Hikosaka et al., 2008).

RANKL is produced by certain types of thymic cells. An initial study revealed that lymphoid tissue inducer cells (LTi) express RANKL in the fetal thymus (Rossi et al., 2007). Furthermore, a recent study uncovered the role of the progenitors of Vγ5 chain-positive dendritic epidermal T cells (DETC), a subset of γδ T cells, in supplying RANKL in the embryonic thymus (Roberts et al., 2012). Interestingly, the RANKL signal in turn induces the expression of Skint-1 in mTECs, promoting the selection and generation of the monoclonal DETC compartment (Barbee et al., 2011), which suggests a role for the mTECs in DETC selection. In addition, RANKL is provided by positively selected α β T cells in adult thymus (Hikosaka et al., 2008).

As described above, because the CD40L expression is practically absent in the fetal thymus, RANKL is essential for the mature mTEC development in embryo (Akiyama et al., 2008). Currently, the relative contributions of LTi and Vγ5^+^ thymocytes to the RANKL expression remain to be determined. Moreover, other lymphoid cells might be involved in the expression of RANKL in the fetal thymus because small numbers of AIRE^+^ mTECs were present in the mice lacking both LTi and Vγ5^+^ thymocytes. In the postnatal thymus, large numbers of α β T cells supply both RANKL and CD40L. Therefore, contributions of LTi and Vγ5^+^ thymocytes might be limited due to their low frequency.

RANKL KO and RANK KO mice did not show any appreciable autoimmune phenotypes, which may be because a small amount of AIRE and low number of mature mTECs are sufficient for suppressing autoimmunity. Another possibility is that the interaction of RANKL and RANK in other immune cells is required for the progression of autoimmunity in peripheral organs. Consistent with the latter possibility, a transplantation of RANK-KO fetal thymic stroma (Rossi et al., 2007) or an adoptive transfer of splenocytes from RANKL-KO or RANKL/CD40 DKO mice (Akiyama et al., 2008) provoked autoimmunity in the recipient nude mice.

## Lymphotoxin β receptor

LtβR is essential for the organogenesis of secondary lymphoid organs (Mebius, 2003; Drayton et al., 2006; Randall et al., 2008). There are at least two types of ligands that bind to LtβR: a heterotrimer consisting of Ltα and Ltβ and the Light homotrimer. Although LtβR-deficient mice show a defect in the development of mTECs (Boehm et al., 2003; Venanzi et al., 2007), the developmental processes regulated by LtβR signaling remain unclear (Zhu et al., 2010). The injection of an agonistic LtβR antibody into mice increases the expression of AIRE within a few hours (Chin et al., 2003), and the treatment of an mTEC line with the same antibody induces the expression of AIRE in the presence of a DNA methylation inhibitor (Chin et al., 2003). However, the expression levels of AIRE in the mTECs isolated from LtβR-KO mice are comparable to wild-type mice (Venanzi et al., 2007), and the treatment of fetal thymic stroma with an agonistic LtβR antibody failed to induce the expression of AIRE (Akiyama et al., 2008). Thus, a direct linkage between LtβR signaling and AIRE expression seems to be unwarranted. It is probable that the LtβR signal controls the development of mTECs by a process i.e., different from that mediated by the RANK and CD40 signals, which are more directly linked to the development of mature AIRE-expressing mTECs. Indeed, LtβR is involved in the expression of AIRE-independent TSAs and chemokines in the thymus (Chin et al., 2006; Zhu et al., 2007; Seach et al., 2008), and a recent study revealed the requirement of LtβR in the development of involucrin-expressing mTECs (White et al., 2010). In addition, another recent study revealed that LtβR signaling induces the expression of RANK, in turn facilitating the development of the mature mTECs (Mouri et al., 2011). These mutually non-exclusive studies suggest that LtβR signaling has multiple roles in the development and functions of mTECs.

LtβR-KO mice exhibit inflammatory cellular infiltration in their peripheral organs (Chin et al., 2006). The fetal thymic stroma transplantation confirmed the requirement of LtβR signaling in the thymic stroma cells for the suppression of autoimmunity (Chin et al., 2006). In addition, the LtβR signal regulates the expression of the chemokines that attract lymphocytes in the medulla (Zhu et al., 2007; Seach et al., 2008). Because the failure in the movement of the positively selected T cells to the medulla results in autoimmunity (Kurobe et al., 2006), this function may be linked to the autoimmune phenotypes observed in LtβR-KO mice.

## Intracellular signal transducers and NF-κB

**Figure 2:**
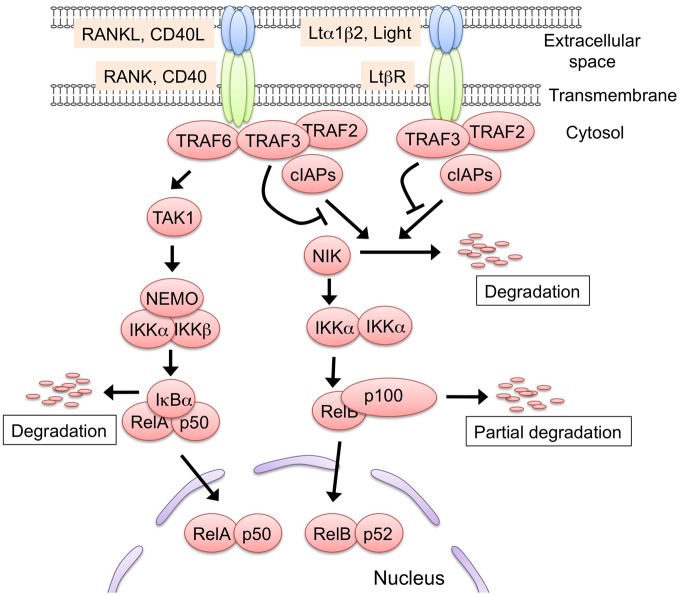
**The two NF-κB activation pathways induced by RANK, CD40, and Ltβ **R** signaling.** Either RANK, CD40, or LtβR signaling is capable of activating the non-classical NF-κB pathway. In the steady state, the protein complex consisting of TRAF2, TRAF3, and cIAP1/2 binds to and poly-ubiquitinates NF-κB inducing kinase (NIK). The poly-ubiquitinated NIK is immediately degraded by the 26S-proteasome machinery. The engagement of the receptors with their ligands recruits the TRAF2, TRAF3, and cIAP1/2 complex, leading to the release of NIK from the complex. The stabilized NIK activates the IKK α complex by phosphorylation, which in turn phosphorylates p100, thus sequestering RelB in the cytoplasm. Subsequently, p100 is poly-ubiquitinated and converted to p52 by partial degradation, an event that leads to the nuclear translocation of the active RelB/p52 complex. The RANKL-RANK and CD40L-CD40 interaction recruits TRAF6 to their cytoplasmic tails. TRAF6 activates the downstream serine/threonine kinase, typically TGF-β activating kinase 1 (TAK1), and activated TAK1 causes the activation of the IKK complex, which consists of IKK α, IKK β, and NEMO. Thereafter, the activated IKK complex phosphorylates IkB α, thereby inducing the degradation of IkB α to lead to the nuclear translocation of the RelA complex. The TRAF6-mediated NF-κB activation might induce the expression of RelB. Although LtβR also can activate the classical pathway, there is no supporting evidence to date that the LtβR-mediated classical pathway regulates mTEC development. Such protein modifications as phosphorylation and ubiquitination are omitted in this figure for simplicity.

RANK, CD40, and LtβR signaling activates two distinct NF-κB activation pathways: the classical and non-classical NF-κB pathways (Hayden and Ghosh, 2008; Vallabhapurapu and Karin, 2009) (Figure 2). In the classical pathway, the engagement of the receptor recruits the TRAF proteins to induce the activation of the downstream serine threonine kinases (e.g., TGF-β activating kinase 1), which in turn activate the IKK complex comprising IKK α, IKK β, and NEMO. Subsequently, the IKK complex phosphorylates IkBα, sequestering NF-κB in the cytoplasm. The phosphorylation of IkBα triggers its degradation by the ubiquitin-dependent 26S-proteasome complex, which in turn results in the translocation of the NF-κB family RelA complex to the nucleus for transcriptional activation. Conversely, in the non-classical pathway, the engagement of the receptors induces the release of NIK from the protein complex comprising cIAP1/2, TRAF2, and TRAF3, a complex that degrades NIK in an unstimulated state. The released NIK phosphorylates IKK α, which in turn phosphorylates the p100 that sequesters the NF-κB family member RelB in the cytoplasm. The phosphorylation of p100 triggers its partial degradation to p52, leading to the nuclear translocation of the RelB/p52 complex to induce gene expression.

RelB-deficient (RelB-KO) mice (Burkly et al., 1995; Weih et al., 1995; Zuklys et al., 2000) and *aly/aly* mice (Miyawaki et al., 1994; Kajiura et al., 2004; Shinzawa et al., 2011), which have a dysfunctional point mutation in the gene encoding NIK, showed severe defects in the development of mTECs expressing AIRE and TSAs. Consistently, these mutant mice exhibit autoimmune phenotypes. Although IKK α-deficient (IKK α-KO) mice die immediately following birth, a defect in mTEC development was still identified (Kinoshita et al., 2006; Lomada et al., 2007). Furthermore, p100 (also named NF-κB2)-deficient (p100-KO) mice showed a partial reduction of the mTEC developmental and autoimmune phenotypes (Zhang et al., 2006; Zhu et al., 2006). Therefore, these data strongly suggested that the non-classical NF-κB pathway is essential for the development of mTECs and mTEC-mediated self-tolerance. Interestingly, whereas the defect in mTEC development is mild in the p100-KO mice, p100 and p105 (also named NF-κB1)-double-deficient mice (Franzoso et al., 1997) and p100 and Bcl-3-double-deficient mice (Zhang et al., 2007) exhibited more severe defects in the development of mTEC compared to each single mutant, suggesting redundant roles among these NF-κB members and the nuclear IκB. Because the non-classical NF-κB pathway can be activated by either RANK, CD40, or LtβR signaling, the developmental stages at which these molecules control mTEC development need to be clarified.

TRAF6, an E3-ubiquitin ligase, activates the classical NF-κB pathway (Inoue et al., 2007), which indirectly facilitates the non-classical NF-κB activation pathway by inducing RelB (Bren et al., 2001; Akiyama et al., 2005) and p100 (Dejardin et al., 2002). Several studies indicated that TRAF6 mediates RANK signaling (Darnay et al., 2007) and CD40 signaling (Bishop et al., 2007) but not LtβR signaling (Qin et al., 2007). Consistently, TRAF6-deficient mice showed a severe defect in mTEC development that was comparable to RANKL and CD40 DKO mice (Akiyama et al., 2005, 2008). Thus, it is likely that, as a downstream molecule of RANK and CD40 signals, TRAF6 is required for the development of mature mTECs expressing AIRE, TSAs, and RelB (Akiyama et al., 2005, 2008).

## Concluding remarks

It is now widely accepted that, despite of the very low frequency in the population of total thymic cells, mTECs play critical roles in preventing autoimmunity in the body. Several studies have provided evidence that RANK, CD40, and LtβR signaling are critical for the development of mTECs. It is most likely that the engagement of these receptors activates the two NF-κB activation pathways. The ensuing transcriptional activation of the NF-κBs induces the genes that promote the development and function of mTECs. However, literature on the downstream target genes induced by these signals in mTECs is currently scarce (Ohshima et al., 2011), and the identification of these targets is important for future research because these target genes would define the unique properties of mTECs. Moreover, the developmental stages of the mTECs receiving this signaling are not fully characterized, and the mechanisms and signals that determine the commitment to the mTEC lineage also remain to be addressed. Future studies, on the biology of mTECs would be informative for the understanding of the mechanism involved in the establishment of self-tolerance and the development of the thymic medullary environment.

### Conflict of interest statement

The authors declare that the research was conducted in the absence of any commercial or financial relationships that could be construed as a potential conflict of interest.
